# Possible roles of exercise and apelin against pregnancy complications

**DOI:** 10.3389/fendo.2022.965167

**Published:** 2022-08-25

**Authors:** Hamed Alizadeh Pahlavani

**Affiliations:** Department of Physical Education, Farhangian University, Tehran, Iran

**Keywords:** exercise, pregnancy, blood pressure, insulin sensitive, myocardial hypertrophy, Preeclampsia

## Abstract

The prevalence of maternal obesity during pregnancy is associated with the risk of gestational diabetes, preeclampsia, and cardiomyopathy. Environmental factors such as active lifestyles and apelin may lead to beneficial changes. In rats, apelin and exercise (45 to 65% VO_2max_ for 6 to 9 weeks) during pregnancy increase brown adipose tissue (BAT) proteins such as Cidea, Elovl3, UCP1, PRDM16, and PGC-1α in males and females fetuses, while white adipose tissue (WAT) is reduced. In humans and animals, apelin and exercise stimulate the expression of the glucose transporters (GLUT1/2/4) in the muscle and adipose tissue through the PI3K/Akt and AMPK pathways. Hence, exercise and apelin may are known as regulators of energy metabolism and be anti-obesity and anti-diabetic properties. In mice, exercise also creates a short-term hypoxic environment in the pregnant mother, activating HIF-1, VEGF, and VEGFR, and increasing angiogenesis. Exercise and apelin also increase vasodilation, angiogenesis, and suppression of inflammation through the L-arginine/eNOS/NO pathway in humans. Exercise can stimulate the ACE2-Ang-(1-7)-Mas axis in parallel with inhibiting the ACE-Ang II-AT1 pathway. Exercise and apelin seem to prevent preeclampsia through these processes. In rats, moderate-intensity exercise (60 to 70% VO_2max_ for 8 weeks) and apelin/APJ also may prevent pathological hypertrophy in pregnancy by activating the PI3K/Akt/mTOR/p70S6K pathway, PI3k-Akt-ERK1/2-p70S6K pathway, and the anti-inflammatory cytokine IL-10. Since pre-clinical studies have been more on animal models, future research with scientific guidelines should pay more attention to human specimens. In future research, time factors such as the first, second, and third trimesters of pregnancy and the intensity and duration of exercise are important variables that should be considered to determine the optimal intensity and duration of exercise.

## Introduction

Pregnancy is a dynamic and organized process for the development of one or more babies (1). Maternal obesity (body mass index (BMI) of 29 kg/m2 or more) has now become a public health concern that may affect the health of mothers and children as well as their longevity (2, 3). Maternal obesity and a sedentary lifestyle also stop the growth of fetal brown fat by impairing its thermogenesis function in the later life phase (4, 5). Common risks associated with maternal obesity in pregnancy include gestational diabetes, vascular disorders such as preeclampsia, and cardiovascular diseases (3, 6–8). Gestational diabetes or insulin resistance during pregnancy is one of the main obstacles to achieving mother and child health. Approximately 9 to 25 percent of pregnancies worldwide are affected by acute long-term complications of the disease (9). On the other hand, preeclampsia is the most common gestational hypertension, affecting 5 to 7% of pregnant women worldwide. Preeclampsia accounts for 20% of all pregnancy deaths, leading to increased preterm birth and fetal growth retardation (1, 10). Cardiomyopathy during pregnancy is the leading cause of non-obstetric mortality. The most common cardiomyopathies in pregnancy are hypertrophic and dilated cardiomyopathy. Hypertrophic cardiomyopathy most often develops one month before or after childbirth, while dilated cardiomyopathy often develops before or during the second trimester (11, 12). Most serious heart accidents (66%) occur in the prenatal period. Almost half of the serious heart events (49%) are preventable and most preventable serious heart events (74%) depend on disease management factors (13). In contrast, numerous studies have suggested moderate-intensity exercise to prevent common pregnancy complications such as maternal obesity, gestational diabetes, vascular disorders, and heart problems in mice. It is reported that exercise (40 to 65% of VO2max for 8 weeks) during pregnancy is beneficial for both mother and fetus to combat obesity in mice (4). In obese pregnant mice, daily exercise on a treadmill reduces weight gain, lowers serum glucose and fat, and increases insulin sensitivity (4, 14). In humans, exercising (40 to 60% of VO2max) during pregnancy reduces the risk of gestational hypertension and preeclampsia and increases energy consumption (15, 16). The fetal and maternal cardiac response to aerobic, anaerobic, and circular exercise activities has been reported to be safe and beneficial during pregnancy in humans and animals (17–19). In general, exercise in obese mothers not only affects the function of fat tissue, but also affects muscle metabolism, glucose homeostasis, preeclampsia, and heart function (4, 14–19). It seems that one of the reasons for these improvements is due to the placentokines such as apelin, leptin, apela, irisin, and adiponectin, but the placentokine we review in this study is apelin. The release of apelin due to exercise also is more than the other placentokines (15). Apelin also is known as exerkine (exercise + cytokine) and placentokine (placenta + cytokine) (15, 20, 21). Apelin as an exerkine reverses obesity-related placental dysfunction by increasing mitochondrial biogenesis in mice (22). It seems that aerobic training (4 times a week with an intensity of 60-70% of the maximum heart rate for 45-60 minutes) affects improving insulin sensitivity through the regulation of apelin (23). A relationship in studies has been reported between swimming (6 days per week for 9 weeks) and apelin expression in rat heart and vessels to reduce blood pressure (24). Hence, exercise (60 to 70% of VO2max, 55 min, 3 days/week, for 8 weeks) may be associated with reduced arterial stiffness by increasing plasma apelin levels in middle-aged adults (25). In addition, exercise (56 to 61% of VO2max, 5 days/week for 3 weeks) may improve myocardial tolerance to doxorubicin-induced cardiotoxicity through apelin by inhibiting oxidative stress and up-regulating antioxidants in rats (26). On the other hand, Apelin precursors and its receptor (APJ) in cattle, humans, rats, and mice show 76 to 95% homology (1). High homology apelin in mammals appears to play similar roles in different species. In pregnant women, the apelin-APJ system changes may promote oxidation of fatty acids, glucose uptake, angiogenesis, vasodilation, hypotension, myocardial contraction, diuresis, and stress response (1). Therefore, in this study, the direct role of apelin through maternal exercise on common pregnancy complications such as obesity, gestational diabetes, gestational hypertension, and cardiac hypertrophy is investigated. This study was conducted to investigate the role of exercise and apelin during pregnancy and also to find effective mechanisms for pregnancy complications such as obesity, gestational diabetes, preeclampsia, and cardiac hypertrophy.

## Apelin and its receptor (APJ)

Apelin is available in various isoforms, including apelin-36, apelin-17, apelin-13, apelin-12, and pyroglutamated [pyr1] apelin-13. Apelin was initially proposed as an endogenous ligand for the angiotensin II protein J (APJ) receptor (27–29). APJ has about 54% homology to angiotensin II receptor (AT1), but angiotensin II cannot bind to it. All apelin isoforms with different biological strengths can bind to APJ (30). Apelin-13 is a high affinity for APJ receptors and apelin-17 and [pyr1] apelin-13 is predominant in plasma (29, 31). Apelin and APJ are present in the muscle, adipocyte, heart, kidneys, and placenta syncytiotrophoblasts (32). Apelin and APJ binding to G protein are expressed in syncytiotrophoblasts for the transport of human placental nutrients (33). Apelin mRNA expression in the placenta is about 10 times that of adipose tissue (34). While apelin in placental villi is 20 times adipose tissue (33). The placenta releases significant levels of apelin, and apelin levels in the fetal circulation are higher than in mothers (1). Decreased levels of apelin in the infant’s circulatory system occur rapidly after birth because the placenta is the source of apelin for the fetus (1). Apelin is highly expressed in the mammary gland during lactation and is positively regulated by the mother’s high-fat diet. Apelin expression in the mammary gland is positively correlated with breast milk apelin and maternal insulin. Apelin levels of breast milk increase with BMI (35). It is reported that apelin and APJ play a role in metabolic diseases, glucose metabolism, atherosclerosis, cardiovascular diseases, oxidative stress, obesity, and pregnancy (32, 36, 37). For example, a negative correlation has been reported between apelin and ox-LDL during pregnancy. In addition, the level of apelin is shown lower in pregnant women, while BMI and serum lipids are significantly high (36). In mice, apelin also increases the level of brown fat proteins, mitochondrial biogenesis, and placental vasculature (22). There is evidence that apelin has a positive effect on fetal BAT development and offspring metabolic health in mice (5). Therefore, apelin appears to be involved in lipid metabolism during pregnancy (38). Apelin has been also reported to be a good candidate for improving insulin sensitivity (31). Studies showed that apelin significantly ameliorates preeclampsia symptoms, impaired endothelial nitric oxide synthase/nitric oxide signaling, and reduces oxidative stress activation in mice (39). In addition, it is reported that apelin is very important for the formation of the fetal cardiovascular system and early placental development, increasing fetal angiogenesis and energy homeostasis in mice (1). Hence, considering the role of apelin in preventing pregnancy complications, in the continuation of the study, we will look at the effects of apelin on markers of pregnancy obesity, gestational diabetes, pregnancy blood pressure, and pregnancy cardiac hypertrophy.

## Association of gestational obesity with white and brown adipose tissue and apelin

Obesity has become an epidemic worldwide due to lack of exercise and overeating, rising from 21.5% to 33.3% in recent years (5, 40). The prevalence of maternal obesity during pregnancy has been reported to be 7-25% and is associated with high birth weight and increases the risk of being overweight in the future (2, 41). Overweight and obesity in pregnant mice predispose their children to obesity and metabolic diseases (42). In obese people, adipose tissue is composed of white adipose tissue (WAT) and brown adipose tissue (BAT). BAT is located in certain places and is the main site of non-shivering thermogenesis in mammals, while WAT collects triglycerides and is responsible for storing metabolic energy (41, 43). WAT reduces metabolic activity due to the lack of thermogenic genes such as PGC-1α and uncoupling protein 1 (UCP1) (44–46), increases lipogenesis during extra calories, and ensures the breakdown of triglycerides (TG) during energy restriction to fuel other organs (46). Increased volume and number of fat cells in WAT is the basis of obesity (47, 48), while brown fat cells lead to decreased due to decreased cellular energy sensor AMP-activated protein kinases (AMPK) and increased inflammatory factors TNF-ɑ (40, 41, 49–51). Hence, maternal obesity in mice impairs the growth of fetal BAT by impairing the thermogenic function of brown fat (5). However, WAT turns into beige or brite cells in response to various stimuli (such as cold and exercise). Beige cells are characterized as thermogenic and brown fat due to high mitochondrial and UCP1 content. While the expression of UCP1 in human WAT is much lower than BAT (52). Therefore, beige cells are found in WAT and burn energy to create heating (53). Browning of beige cells occurs in response to various stimuli such as exposure to chronic cold, caloric restriction, exercise, maternal lifestyle, nutrition, and apelin administration (41, 52). Brown and beige fat cells use free fatty acids (FFA) for mitochondrial beta-oxidation (46, 53). Enhancing the thermogenic function of BAT and beige fat cells eliminates excess energy and leads to the prevention of obesity and metabolic diseases (53). In obesity, apelin appears to act to inhibit adipogenesis through negative feedback at the autocrine level, as obesity increases apelin and APJ secretion. While the inhibition of apelin leads to obesity and obesity increases with the lack of apelin signaling (54). In other words, in obesity, the formation of brown fat is suppressed and the reduction of excess energy consumption in WAT is intensified. On the other hand, apelin expression is increased in WAT and its plasma level is increased in obesity. Apelin activates PI3K/Akt and AMPK signaling in brown preadipocytes (52). Hence, apelin creates a positive feedback loop to increase the differentiation of brown fat cells and neutralize the adipogenesis of brown disrupted by inflammatory agents. It has been reported that the weight of WAT is significantly reduced in apelin-treated rats, while the weight of BAT is similar to that of the control group. However, the level of brown fat marker proteins in WAT (PRDM16, COX1, and UCP1) increases in mice treated with apelin (52). Hence, apelin is known as a regulator of energy metabolism and has anti-obesity and anti-diabetic properties (52).

## The possible effect of apelin on adipose tissue differentiation in obese pregnant women

In rats, apelin mRNA in WAT increases 2.2-fold on day 7 of gestation and returned to baseline on day 14, and remained at baseline during pregnancy (55). Apelin mRNA expression in BAT during pregnancy and lactation is similar to WAT. The reason for the increase in early pregnancy may be related to fat accumulation (55). Hence, appears apelin mRNA levels and apelin expression are increased during the differentiation of fat cells (56, 57). On the other hand, plasma apelin levels are significantly higher in obese patients than in normal individuals (56). In addition, plasma apelin, placental apelin, and APJ expression all increase in obese pregnant rodents (33, 58, 59). However, it is reported that plasma apelin is no different in obese women (BMI = 35.8) with heavy infants than in normal women (BMI = 23.1) with normal-weight infants (33). It has also been reported that in obese mice, the expression of apelin and APJ in the placenta increases, and the concentration of apelin in the mammary glands is higher in obese women and insulin-resistant obese women than in the control group. Also, more APJ mRNA expression was reported in obese women than in normal women. While maternal and neonatal apelin secretion is reduced in obesity and insulin-resistant obesity compared with controls (59). These differences in the level of the apelin are due to the time, dose, and place of measurement during pregnancy. But to answer these differences, it is better to focus on the function of the apelin on adipose tissue.

**Figure 1:**
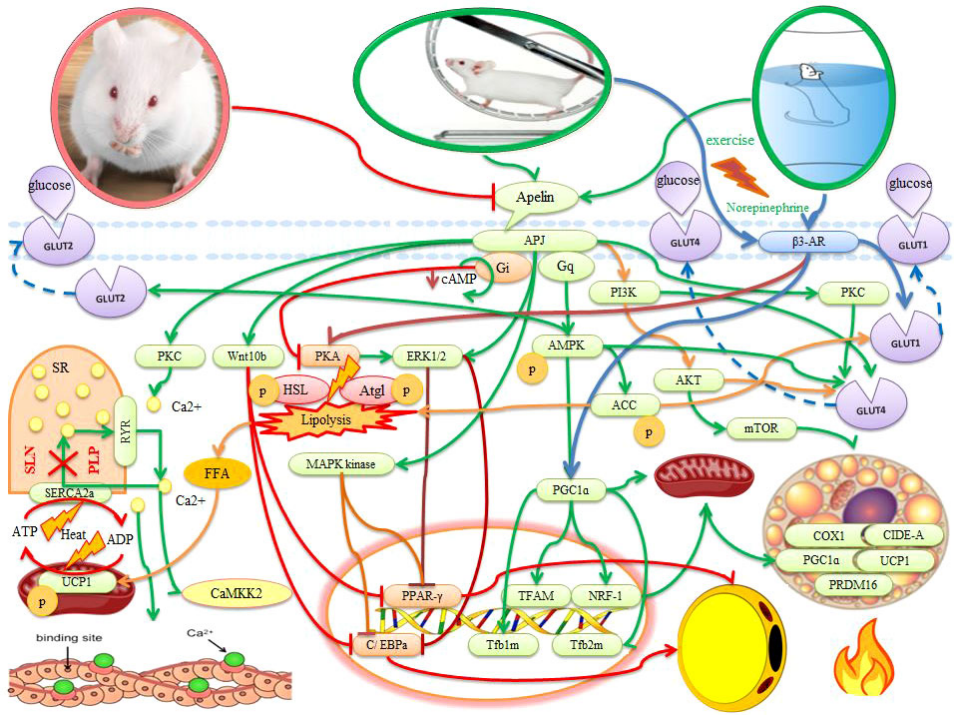
The effects of apelin and exercise on adipose tissue, muscle, and placenta in animals. The description is available in the text. Abbreviations are given at the beginning of the study.

Apelin stimulates transcription factors such as CCAAT-enhancer-binding protein (C/EBPβ) and peroxisome-proliferator-activated receptor-γ (PPARγ) in the early stages of adipocytes differentiation. While apelin increases transcription factors such as PR domain-containing protein 16 (PRDM16) and peroxisome proliferator-activated receptor-gamma coactivator 1-alpha (PGC1α) for mitochondrial biogenesis and thermogenesis (40, 52, 54). Brown cell adipogenesis is stimulated by the binding of PRDM16 to PPAR-γ and activation of the transcriptional function (60). Apelin then counteracts PPARγ to increase brown fat in the next stage of differentiation. Hence, C/EBPβ and PPARγ are critical for the initiation of WAT and BAT production, while PRDM16 and PGC1α are essential for the determination of BAT. Hence, it seems that increasing apelin is to increase BAT activity and counteracts WAT (40, 52, 54) (**Figure 1**). Apelin increases the expression of PRDM16 in WAT and leads to the browning of WAT (52). In addition, lack of PRDM16 in brown fat precursors leads to loss of brown fat characteristics, and overexpression of PRDM16 in myoblasts causes them to differentiate into brown fat cells (60). Expression of UCP1, PRDM16, and COX1 also significantly increase in human WAT treated with apelin on days 5-9 (52). Apelin-APJ signaling also differentiates BAT through the PI3K/Akt and AMPK/mTOR signaling pathways and increases the expression of brown fat proteins for mitochondrial biogenesis and thermogenesis (UCP1, CIDE-A, and COX1) (52) (**Figure 1**). Therefore, PRDM16, UCP1, CIDE-A, and COX1 gene expression can be a key strategy to reduce metabolic disorders caused by obesity because brown fat can increase energy consumption and protect against obesity through a specialized energy-burning program (5, 52, 60). Hence, in childhood and adolescence, BAT plays an important role in metabolic processes such as glucose and lipid metabolism (41). Apelin also has been reported to reduce levels of WAT, serum triglycerides, FFA and glycerol release, abdominal obesity, fat weight, and fat production in obese rats. While mice with apelin deficiency increase serum free fatty acid levels, serum FFA and glycerol levels, abdominal obesity, and body fat (52, 61). In rats, apelin can also significantly reduce TG content, glycerol concentration, the average diameter and adipocytes, and expression of PPARγ and perilipin mRNA in WAT. Apelin can inhibit fat cell differentiation, enhance lipolysis and improve obesity. Possible mechanisms may be to regulate PPARγ expression to inhibit WAT differentiation and to regulate perilipin expression to promote lipolysis (40). Hence the increase in apelin in obesity seems natural because autocrine apelin signaling may serve as a new therapeutic target for obesity and other metabolic disorders (54).

It is reported that daily apelin supplementation during pregnancy results in an increase in BAT marker proteins such as UCP1, PRDM16, and PGC-1α in mice fetal. Apelin administration also induces the expression of UCP1, Ppargc1a, PRDM16, Cidea (BAT-specific marker), and Elovl3 mRNAs in male and female fetuses (5) (**Figure 1**). In addition, Increased expression of UCP1 regulates many of the genes involved in lipid metabolism in BAT (41). In BAT, UCP1 is activated by FFA, causing oxidative phosphorylation to separate from ATP production as a proton carrier. UCP1 as a proton channel also disperses the electrochemical gradient without ATP production. Therefore, to adapt to the inefficient production of ATP, metabolism must be increased and heat produced. Hence, lipid catabolism in BAT does not lead to the production of ATP but also thermogenic reactions. Fatty acids released from fat droplets are the primary energy source for UCP1-mediated thermogenesis in BAT (46). Glucose metabolism is very important for BAT activity for lipogenesis or filling of fat droplets and can be used as an alternative fuel (46). These effects of apelin on WAT and BAT indicate the use of energy reserves and the reduction of adipose tissue (2). In addition, apelin increases the basal activity of BAT by increasing the expression of PGC1α, UCP1, mitochondrial biogenesis, and oxygen consumption (52). Thus, positive regulation of thermogenic genes in fetal BAT may be a strategy for the use of fat storages that can be used immediately by infants for UCP1-mediated thermogenic after birth (62). Apelin also has the potential to increase oxygen consumption, and reduce oxidative stress in fat cells, leading to anti-apoptotic effects. The anti-apoptotic effect of apelin in different cells is exerted by mitogen-activated protein kinase (ERK1/2, MAP3/1) and protein kinase B (AKT) (5, 52, 63–65). Therefore, the administration of apelin during pregnancy both increases brown lipid production, oxidative phosphorylation, and mitochondrial activity and inhibits the process of lipid synthesis and differentiation of white fat cells in fetal BAT (5). Thus, apelin can be a new therapeutic target for obesity and metabolic diseases in children of obese mothers (2, 52).

## Possible mechanisms of apelin in the adipose tissue

It is reported that apelin administration stimulates AMPK activation and increases DNA demethylation of the PRDM16 promoter, leading to the initiation and maintenance of BAT production and thermogenesis (5). There is evidence also that apelin in pre-adipocytes and adipocytes leads to phosphorylation of MAPK kinase/ERK1/2 and inhibition of expression of adipogenic transcription factors (PPARγ and C/EBPa) (54). In addition, apelin through Wnt (Wnt10b) stimulation may inhibit the expression of PPARγ and C/EBPa in the early stages of differentiation. As a result, this event inhibits adipogenesis (54) (**Figure 1**). In adipocytes, apelin binds to the APJ and activates G protein subunits that include the Gαq and Gai proteins. Gαq and Gai then activate AMPK and inhibit protein kinase A (PKA) and reduce cyclic adenosine monophosphate (cAMP) synthesis. Hence, apelin inhibits lipolysis through Gq-AMPK phosphorylation and Gi-PKA dephosphorylation (61) (**Figure 1**). Apelin also performs ACC phosphorylation through AMPK activation An increase in apelin-induced lipogenic enzymes such as ACC leads to an increase in FFA synthesis in BAT, while FFA synthesis decrease in WAT. However, the FA content decreases significantly in BAT because the rate of decomposition of FFA (e.g., β-oxidation) also increases. This represents a collaboration between WAT and BAT for thermogenesis (66). Apelin also inhibits lipolysis by preventing the fragmentation of lipid droplets, increasing the expression of AMP-dependent perilipin, and decreasing the phosphorylation of perilipin. Therefore, the decrease in the release of free fatty acids caused by apelin can be attributed to the dual function of apelin in lipogenesis inhibition and lipolysis inhibition. Hence, apelin and APJ have been shown to inhibit adipogenesis of pre-adipocytes and lipolysis in mature adipocytes (54). Daily injections of apelin have also been reported to reduce triglyceride content, fat deposition in adipose tissue, and the expression of a variety of genes involved in adipogenesis while increasing thermogenesis and oxygen consumption (40, 52). Since most of these findings are based on animal models, more attention should be paid to human models. The study of these key variables and important pathways in different time and place conditions in pregnant women should be considered.

## Possible roles of exercise and apelin on adipose tissue of pregnant women

**Figure 2:**
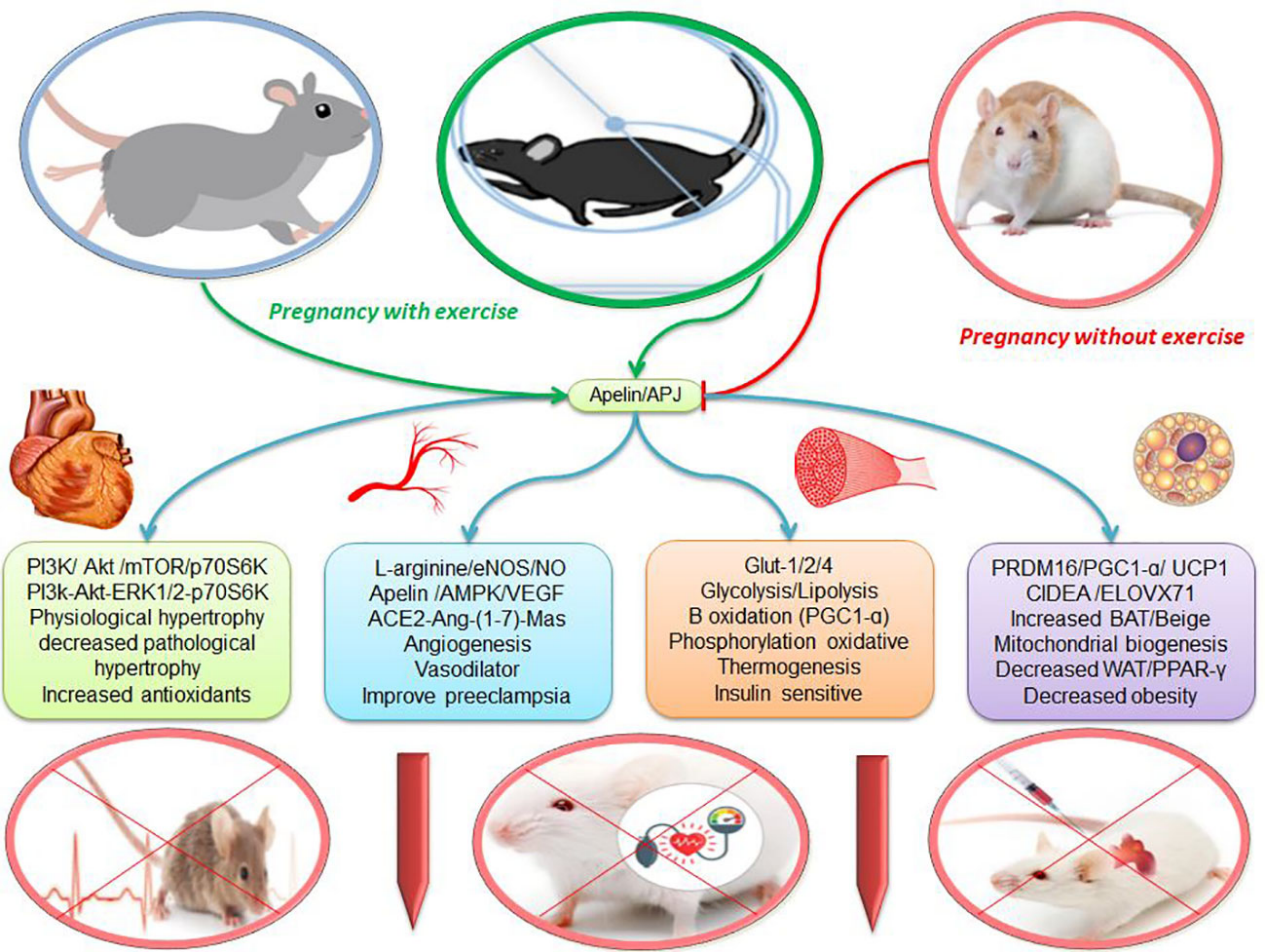
The effects of apelin and exercise on adipose tissue, muscle, vascular and heart. escription is available in the text. Abbreviations are given at the beginning of the study.

Among the increase in hormones caused by maternal exercise, apelin has a higher level because it is present in large amounts in the circulation of the mother, fetus, and placenta (41). Exercise-induced placental hypoxia stimulates vascular proliferation and expression of apelin in the placenta, increased apelin in fetal circulation, increased apelin in maternal circulation, and increases fetal brown fat (5). In mice, exercise (45 to 65% VO2max for 8 weeks) positively regulate apelin and is maintained in their offspring after a high-fat diet. In fetal brown adipose tissue, protein levels of UCP1, PRDM16, and PGC-1α are higher in athlete mothers than in controls. In the offspring of athlete mothers *via* apelin, the mRNA expression of BAT markers including UCP1, Ppargc1a, and PRDM16 increases (5) (**Figure 2**). In addition to the above factors, exercise increases the protein content of BAT markers such as Cidea and Elovl3 in male and female fetuses (5, 22). On the other hand, sedentary, overweight adults increase UCP1 by 12 weeks of exercise (3 times per week, HRmax intensity 70-80%). As a result, it appears that 12 weeks of exercise with apelin changes leads to the expression of brown/beige fat genes in abdominal fat (67). However, after swimming the pregnant mother is not reported any difference in the expression of UCP1 in BAT, which is related to the type of exercise because swimming puts less pressure on the athlete’s mother due to carrying part of the body weight in water (2). In addition, pregnancy obesity decreases PRDM16 expression, oxidative metabolism, and mitochondrial biogenesis in the placenta. while maternal exercise-induced administration of apelin and apelin significantly increases PRDM16 expression and is associated with increased oxidative metabolism and mitochondrial biogenesis and expression of nutrient transporter in the placenta (22). Exercise increases BAT markers such as UCP1 and PRDM16 in the offspring of athlete mothers and is associated with oxygen consumption (**Figure 2**). In mice following a high-fat diet, VO2 and VCO2 levels are higher in the offspring of athlete mothers than in the control group. Thus, maternal exercise increases carbohydrate oxidation in the offspring of athlete mothers who are challenged with a high-fat diet. Increased oxygen consumption and carbohydrate oxidation are associated with increased weight and BAT temperature in the offspring of athlete mothers (5). Then, WAT weight is reduced for the offspring of athletic mothers and they show better glucose tolerance than the offspring of the control group, which is associated with lower levels of fasting blood sugar and insulin levels in the offspring of athlete mothers (5). There is a negative correlation between UCP1 protein levels and insulin resistance in children of mothers of athletes exposed to high-fat diets, indicating that maternal exercise protects against metabolic disorders from children with high-fat diets. Exercise of the pregnant mother seems to increase the growth of beige/BAT fat in the fetus and children against obesity and metabolic syndromes (5, 41). Maternal exercise (45 to 65% of VO2max for 8 weeks) via apelin reduces pregnancy weight gain and increases the relative weight of BAT by 37.8%, while WAT decreases by about 37.1% compared to control mice. During weaning, body weight and WAT mass are lower in the offspring of athlete mothers, but WAT mass is higher than in the offspring of sedentary mice. Children of athletic mothers consume more food than children in the control group after exposure to a high-fat diet. However, the offspring of athlete mothers show less weight gain than the control group (2, 5). Therefore, exercise and consumption of apelin by the pregnant mother regulates glucose and fat metabolism, oxidative phosphorylation, thermogenesis, mitochondrial biogenesis, and growth of fetal brown fat and prevents the differentiation of white fat cells in fetal brown fat (5, 68).

In rodents, sympathetic stimulation using external stimuli (such as exercise, norepinephrine, prolonged cold exposure, and the PPARγ agonist) activates the beta-adrenergic receptor 3 (β3-AR) (69). In rats, exercise (60% of VO2max for 6 to 9 weeks) also directly activates the central nervous system (SNS) and, releasing norepinephrine and through β3-AR, stimulates BAT activity and WAT browning (70–72). In other words, the SNS innervates adipose tissue and releases the hormone norepinephrine. Norepinephrine stimulates β3-AR in fat cells and leads to an increase in cAMP. The cAMP-dependent PKA is then activated. PKA regulates phosphorylation of lipolytic enzymes and increases access to substrates for thermogenic and UCP1 activation (70) (**Figure 1**). Increased PKA activation can then phosphorylate hormone-sensitive lipase (HSL), adipose triglyceride lipase (Atgl), and perilipin, thereby activating lipolysis. Activation of PKA causes the excretion of the substrate (FFA) for thermogenesis (69, 72) (**Figure 1**). The primary source of thermogenic energy in BAT is also FA because it is essential for activating UCP1. BAT thermogenesis appears to be strongly associated with TG hydrolysis and FA oxidation (73). In addition, glucose appears to be used to regenerate ATP in mitochondria *via* UCP1 because lactic acid (an indicator of anaerobic glycolysis) increases significantly with thermogenesis. On the other hand, UCP1-induced thermogenesis impairs BAT thermogenesis by inhibiting glycolysis because increased glycolysis is also necessary to provide sufficient oxaloacetate for the oxidation of FA and acetyl CoA in the citric acid cycle (73–75). Therefore, carbohydrates and fats are consumed in BAT, and heat production prevents obesity. While people with low body temperature or low thermogenesis are more prone to obesity (76). On the other hand, norepinephrine also activates the expression of PGC1α (a key regulator of thermogenic gene expression and mitochondrial biogenesis), COX1, and UCP1 *via* β3-AR receptors. It then increases mitochondrial biogenesis, oxygen uptake and respiratory activity, and metabolic rate in BAT. It seems that increased apelin with exercise can be used as a new therapeutic target for obesity and metabolic diseases (52). Because healthy obese pregnant women have the aerobic capacity for physical activity, it is recommended that obese pregnant women exercise three to four times a week (repetition) and walk 10,000 steps daily (77).

## Possible roles of exercise and apelin for skeletal muscle thermogenesis in pregnant women

In rats, the concentration of apelin on the 21st day of pregnancy rises significantly and causes uterine contractions. These contractions do not occur with the protein kinase C (PKC) inhibitor in a calcium-free environment, suggesting that the PKC pathway may be involved in the apelin mechanism (78). This indicates the importance of calcium in muscle contractions and thermogenesis. In myocytes, SERCA is located in the sarcoplasmic reticulum (SR) membrane and transports calcium from the cytosol to the SR using ATP hydrolysis. The gradient of calcium produced by SERCA is dispersed by the ryanodine receptor (RyR1). SERCA transport activity is inhibited by the two peptides phospholamban (PLP) or sarcolipin (SLN), but its ATPase activity remains. Thus, to match Ca2 + transport, mitochondrial ATP synthesis is increased and heat is generated (46) (**Figure 1**). Under conditions of hyperthermia, there is uncontrollable Ca2+ leakage with stimulation of the calcium cycle without muscle contraction and heat production (73, 79). It is reported that exercise-induced apelin in mothers activates sarcolipin and uncoupling protein 3 (UCP3) in the muscles of children and protects them from obesity (2) because Sarcolipin and UCP3 regulate thermogenesis in muscles to improve metabolic homeostasis (42). In myogenic cells with apelin/APJ deficiency, a decrease in thermogenic genes (Sarcolipin and UCP3) indicates the effect of apelin/APJ on fetal muscle thermogenesis and lower muscle temperature (2). After exercise, apelin activates Gαi and Gαq. Apelin activates CaMKK2 (calcium/calmodulin-dependent protein kinase kinase 2) as a downstream Gαq molecule in the fetal muscle of obese mothers and stimulates fetal muscle thermogenesis (42). In the fetal muscle of obese mothers, CaMKK2 is inactivated while it is regenerated by apelin administration. Apelin mimics the effects of exercise and increases the thermogenic capacity of fetal muscles (2). Apelin also activates AMPK as a mechanical mediator in the regulation of metabolic pathways. AMPK ablation reduces the beneficial effect of exercise-induced apelin on fetal muscle thermogenic and mitochondrial biogenesis genes (2, 80). Hence, the heat generated by apelin may be due to the activation of AMPK (5, 61, 80). Activation of AMPK through exercise increases the PGC1-α level and its downstream (NRF1 and TFAM) for proliferation and transcription of mitochondrial DNA. PGC-1α stimulates mitochondrial biogenesis and regenerates mitochondrial-rich oxidative muscle tissue to regulate carbohydrate and fat metabolism. Hence, exercise-induced PGC-1α may play a role in improving obesity and type 2 diabetes (80, 81). There is evidence that a high-fat diet of parents through hypermethylation of the PGC-1α promoter leads to impaired metabolic homeostasis in boys at 9 months of age (82). In addition, maternal obesity lower fetal muscle temperature and thermogenic markers while being restored by maternal exercise (2). Thus, exercise prevents child obesity due to maternal obesity, which currently accounts for approximately 35% of pregnancies (42). In summary, maternal obesity is inhibited by exercise through activation of AMPK, PGC-1α, UCP3, and sarcolipin in fetal muscle. It is noteworthy that these changes are maintained in the muscles of children and show intergenerational effects (42). In general, it appears that exercise and apelin can increase thermogenesis in WAT, BAT, beige, and muscle, and these processes can lead to reduced obesity and metabolic disorders.

## Possible effects of apelin on insulin sensitivity in pregnancy

Gestational diabetes is a glucose intolerance during pregnancy and its lack of control leads to side effects such as birth defects (31). Gestational diabetes has several short-term and long-term complications in both mother and fetus, including preeclampsia, fetal macrosomia or type 2 diabetes, and cardiovascular disease in the future (1). Nevertheless, the level of apelin in pregnancy with and without diabetes is controversial. For example, no association has been reported between plasma apelin concentrations in gestational diabetes and controls (83). The concentration of apelin also in cord blood has not different reported in diabetics and normal people (31). In addition, some studies have reported no significant difference between women with gestational diabetes and women with normal glucose tolerance at plasma apelin mRNA levels as well as apelin and APJ mRNA expression in adipose tissue, visceral fat, and placental tissue (34). But other studies about the apelin effect in other tissue have reported differently. For example, it has been reported that circulating levels of apelin, an insulin-sensitizing hormone, in the placenta increase in mothers of obese and insulin-resistant pregnant mice (1, 33). It also is reported that in obese patients, plasma apelin and insulin levels are significantly higher and the expression of apelin in fat cells is strongly inhibited by fasting and improves after re-feeding like insulin (56). This means that insulin deficiency increases the concentration of apelin and apelin can act like insulin (31). There is also evidence that the level of apelin in the group of fasting pregnant women is significantly higher than non-fasting pregnant women, which is may be related to insulin resistance at they (84). While severe maternal food restriction during pregnancy reduces fetal apelin, indicating that maternal nutritional and glucose status affects fetal apelin levels (1). Also, maternal plasma apelin levels have been reported to be significantly higher in patients with gestational diabetes compared to pregnant women without diabetes, which is positively correlated with their umbilical cord blood levels (83). In the second trimester, apelin levels also are reported to be higher in patients with gestational diabetes than in controls, with a negative correlation with triglycerides (TG) and total cholesterol (38). In addition, it is reported in pregnant women (24 to 28 weeks of gestation) insulin levels are significantly higher than in non-pregnant women, while serum apelin and glucose levels are lower. Nevertheless, there was a negative correlation between apelin with ox-LDL and HDL-cholesterol in the pregnancy group (36, 85). Thus, apelin appears to be involved in glucose and lipid metabolism during pregnancy. One of the reasons for these differences is mostly due to measurement methods that have used real-time PCR, Western blot, and ELISA methods. On the other hand, feeding conditions, time, and place of measurement can affect these differences. In the following, we will discuss the effects of apelin in gestational diabetes.

Apelin increases pancreatic islet cell mass and beta-cell insulin content in mice (86). Apelin has also a glucose-lowering effect along with increased glucose utilization in skeletal muscle and adipose tissue of normal and obese insulin-resistant mice (34). Administration of low-dose apelin to the fetus on day 21 of the fetus leads to the uptake of glucose into the lungs and muscles of the fetus in an insulin-independent manner. Administration of apelin at higher concentrations decreases fetal insulin and increases fetal glycemia, indicating the inhibitory role of high plasma apelin levels in insulin secretion in the fetus (1). In addition, short- and long-term treatment with apelin improves insulin sensitivity in obese and insulin-resistant rodents by increasing glucose uptake into skeletal muscle (86). Apelin increases glucose uptake *via* insulin and the PI3K/Akt pathway in adipocytes and can increase the transport of glucose transporter 4 (GLUT4) from the cytoplasm to the plasma membrane (87) (**Figure 1**). Endoplasmic reticulum stress-induced diabetes is ameliorated by oral apelin-13 administration by inhibition of inositol-requiring enzyme 1α (IRE1α) and inhibition of c-Jun N-terminal Kinase (JNK) (31). Direct regulation of apelin expression by insulin in both human and mouse adipocytes is associated with the stimulation of phosphatidylinositol 3-kinase (PI3K), PKC, and MAPK. Thus, insulin has direct control over the expression of the apelin gene in adipocytes (56). In diabetes or obesity with hyperinsulinemia, apelin levels increase. Hyperapelinemia is a compensatory mechanism that not only inhibits pancreatic secretion but also leads to insulin sensitivity and glucose uptake into insulin-independent muscle tissue in mice (31). In conclusion, the apelin/APJ axis appears to be involved in the regulation of glucose homeostasis in adults by stimulating glucose uptake and insulin sensitivity (1). Increased apelin is believed to be a compensatory mechanism for inhibiting pancreatic secretion, insulin sensitivity, and glucose uptake into insulin-independent muscle tissue (31). Increased plasma apelin in type 2 and type 1 diabetic patients confirms the existence of this compensatory mechanism, which first reduces insulin resistance and then leads to a decrease in apelin levels. These low serum apelin levels in healthy lean individuals may be the result of natural insulin sensitivity (31, 88). Thus, apelin also has anti-diabetic properties and can be used as a therapeutic agent for type I and II diabetes (89). To understand these issues, we need to pay more attention to these apelin mechanisms in pregnancy in future research.

Apelin stimulates the phosphorylation of AMPK and acetyl CoA carboxylase (ACC) in muscle (68, 90). Chronic apelin therapy increases GLUT2 by activating AMPK and enhances glucose uptake and utilization of glucose in skeletal muscle in obese and insulin-resistant mice (31, 91) (**Figure 1**). On the other hand, the abundance of apelin and APJ in the placenta is strongly related to placental AMPK signaling and causes the transfer of maternal glucose to the fetus because all effects in mice treated with apelin are eliminated by inactivating AMPK (33, 92). In addition, during AMPK activation, ACC is inhibited, then the concentration of malonyl-coenzyme A (CoA) decreases, and beta-oxidation of fatty acids increases in muscle (15, 93, 94). In the human placenta, ACC is phosphorylated *via* p-AMPK to enhance fatty acid oxidation and reduce fatty acid synthesis (95). While ACC phosphorylation in the diabetic macrosomic group is significantly inhibited and leads to macrosomic induction through an excessive synthesis of fatty acids in the placenta (95). In the macrosomic diabetic placenta group, there is a significant negative correlation between neonatal birth weight and p-ACC and p-AMPK protein expression (95) (**Figure 1**). This means that the levels of the GLUT1 and ACC proteins increase and the p-AMPKα and p-ACC protein levels decrease in the macrosomia of gestational diabetes. As a result, GLUT1 has a potential role in the transfer of placental glucose to infant weight in gestational diabetes, which is the reason for the significant increase in macrosomia due to gestational diabetes in late pregnancy (95). Obese and insulin-resistant mice make better use of lipids, oxidation of free fatty acids, oxidative capacity, and mitochondrial biogenesis in muscle by administering apelin. Hence, injections of apelin for 4 weeks reduce fat mass, glycemic levels, and plasma triglycerides (92). Therefore, apelin can improve insulin sensitivity by increasing glucose uptake, mitochondrial biogenesis, improving oxidative capacity, and full use of lipids in insulin-resistant mice (68, 92).

## The effect of exercise on insulin sensitivity in pregnancy

Exercise results on gestational diabetes are challenging. For example, it was reported that there was no difference between a standard 12-week exercise program or standard prenatal care (control group) in the second half of pregnancy to prevent insulin resistance. This study has several drawbacks in implementation. These drawbacks include determining the intensity of exercise (Borg scale), low adherence of participants, exercising at home without supervision, and doing exercise in the control group. These flaws make the results of the study questionable (96, 97). Some other findings support these findings that exercise has no significant effect on gestational diabetes (98, 99). In contrast, other studies report different results, for example, exercise reduces the risk of gestational diabetes. This study states that to achieve at least a 25% reduction in gestational diabetes, pregnant women should exercise at least 600 MET-minutes per week (e.g., 140 minutes of brisk walking, water aerobics, stationary cycling, or resistance training) with moderate intensity (100). There is evidence that obese women with 30 minutes of cycling three times a week (from the 25th week of pregnancy to the end of pregnancy) gain significantly less weight and lower levels of insulin resistance (101). There is evidence that women with gestational diabetes with exercise intervention have the lowest BMI increase in late and mid-pregnancy compared to women with gestational diabetes without exercise intervention. In addition, athletic diabetic women experience a lower risk of preterm delivery, high birth weight, and macrosomia than non-athletic diabetic women (97, 98, 102–104). Studies also show that for some women, exercise may help reduce the risk of gestational diabetes. Therefore, exercise seems to be associated with a reduction in gestational diabetes in obese women (105, 106). Therefore, moderate-intensity exercise in the second and third trimesters of pregnancy can be used to reduce the significant adverse outcomes of gestational diabetes (107–109). The possible mechanisms of these important effects of exercise in gestational diabetes can also be important. Some of these mechanisms are mentioned below.

## Possible mechanisms of exercise on gestational diabetes

After exercise, increased expression of UCP1 protein in BAT and WAT in the offspring of exercise mothers compared to control rats leads to improved glucose tolerance, insulin sensitivity, decreased fasting glucose, and insulin levels (5). After exercise, BAT also clears and absorbs glucose through two different pathways: SNS stimulation and insulin signaling. These two pathways have a synergistic effect on the transfer of glucose transporter to the plasma membrane. In BAT, stimulation of β3-adrenoceptors by epinephrine and norepinephrine increases the expression of GLUT1 and GLUT4 to the plasma membrane. Glucose uptake after adrenergic stimulation is independent of GLUT4 and is dependent on GLUT1 displacement. In BAT, however, insulin enhances GLUT4 transport to the plasma membrane *via* the PI3K-PDK-Akt pathway (**Figure 1**). Activation of BAT by adrenergic stimulation can reduce high cholesterol and atherosclerosis and regulate lipid metabolism in humans. Hence, brown fat volume is associated with increased lipolysis, free fatty acid oxidation, and insulin sensitivity (41, 110). Therefore, BAT is a major organ for burning fatty acids and glucose for thermogenesis (111).

Exercise-induced apelin secretion by activation of AMPK stimulates the expression of the GLUT4 gene in muscle (93). Hence, there is a significant positive correlation between apelin after exercise and decreased insulin resistance (112). In diabetic mothers, exercise-induced apelin has preventive effects on gestational diabetes by reducing insulin and glucose in maternal circulation, reducing weight, reducing insulin resistance, and improving glucose tolerance in skeletal muscles (2, 5). Apelin from exercise (40 to 60% of VO2max for 1 week) in fetal skeletal muscle activates AMPK for mitochondrial biogenesis by binding to a G protein-coupled receptor. The number of mitochondrial DNA (mtDNA) copies in the muscle of male and female fetuses increase (15, 91). Maternal exercise and consumption of apelin during pregnancy cause DNA demethylation of the peroxisome proliferator-activated receptor γ coactivator-1α (Ppargc1a) promoter and increase mitochondrial biogenesis in fetal muscle (15, 91). After exercise, APJ severely induces mitochondrial biogenesis markers such as Ppargc1a, Tfam, Tfb1m, Tfb2m, Nrf1, and Cox7a1 in males and females myogenic cells (15, 91) (**Figure 1**). These changes are maintained in the offspring and the offspring muscle in athlete mothers express higher levels of PGC-1α1/4 isoforms. Taken together, these effects have a positive effect on children’s endurance capacity and protect children’s muscles against metabolic disorders (15, 91). Treatment of mice with insulin resistance by apelin not only improves insulin sensitivity, but also increases fatty acid oxidation, oxidative phosphorylation, and mitochondrial biogenesis (31). Exercise of the pregnant mother also increases oxidative muscle fibers in the offspring by increasing mitochondrial biogenesis in oxidative fibers compared to glycolytic fibers. Oxidative muscle fibers are very efficient at using glucose and fatty acids (91). Hence, the useless energy consumption in the muscle increases while the thermogenic function is disrupted due to obesity caused by high energy nutrition. Therefore, exercise along with thermogenic function in muscle prevents metabolic disorders such as obesity and insulin sensitivity (2, 42). In addition, the sons and daughters of athletic mothers show better glucose tolerance than the children of the control group, which is associated with lower fasting blood sugar and insulin levels in the offspring of athletic mothers (5). These data show the long-term beneficial effects of maternal exercise on the metabolic health of children and support the preventive role of maternal exercise in glucose tolerance and metabolism of athlete children.

## Preeclampsia and pregnancy

Hypertension is a very common risk factor for cardiovascular disease (CVD) (113). Women are more likely than men to die from cardiovascular disease, as well as deaths from cardiovascular disease are on the rise for women under 55 years (114). Around the world, about 7 million women are diagnosed with pregnancy hypertension or preeclampsia every year, and it usually appears in the third trimester of pregnancy (after 20 weeks of pregnancy) and is one of the main causes of maternal, fetal, and neonatal mortality (1, 115, 116). Premature birth between 32 and 34 weeks of gestation is very high due to the risk of discomfort to the mother and fetus due to preeclampsia (1). In addition, low birth weight is a recurrent consequence of preeclampsia and is independently associated with increased blood pressure and ischemic heart disease in adulthood (115, 116). This process is because embryonic development is based on the formation of the placenta because it creates conditions for the exchange of nutrients and oxygen between mother and fetus (1). In addition, the initial events of preeclampsia appear to be still placental ischemia/hypoxia. One of the main causes of placental ischemia is insufficient trophoblast invasion, which results in incomplete regeneration of the uterine spiral arteries and endothelial vasospasm (32). Hence, symptoms of preeclampsia include endothelial dysfunction such as vasoconstriction and ischemia (1). Invasion of embryonic trophoblast cells into spiral arteries reduces the resistance of placental arteries and increases blood flow to the embryo-placenta unit (117). However, women and children who survive preeclampsia are at greater risk for future cardiovascular adverse events (115, 116). That is why it is vital to address this issue.

## Preeclampsia and the possible roles of apelin and exercise

Several mechanisms are associated with endothelial dysfunction in preeclampsia. These include hypoxia, excessive oxidative stress, renin-aldosterone-angiotensin (RAS) axis, and imbalance of placental angiogenic factors. This means that the soluble fms-like tyrosine kinase 1 (sFIt-1), as an anti-angiogenic factor, binds to the vascular endothelial growth factor receptor (VEGFR) and neutralizes vascular endothelial growth factor (VEGF) and placental growth factor (114). In addition, preeclampsia is associated with an increase in placental anti-angiogenic agents (sFlt1 or sVEGFR-1) and endoglin because the administration of these agents to pregnant mice causes preeclampsia (1). Recent studies have also identified decreased apelin and APJ as important factors in preeclampsia at the placentas (32, 39, 118, 119). Apelin mRNA is in mature fat cells and stroma vascular fraction (SVF) at approximately equal levels (56). Inefficient Apelin and APJ appear to help initiate preeclampsia by reducing angiogenic activity in the placenta (32, 119). Apelin is important for the formation of the fetal cardiovascular system and the early growth of the placenta and acts in mid or late pregnancy to modulate fetal angiogenesis (1, 39). The role of apelin and APJ in early pregnancy is likely because stronger APJ signals are seen in trophoblast cell membranes (119).

On the other hand, apelin is known to be a potent mediator for placental arteries, and the content of apelin decreases as a result of high-fat diets but is positively regulated after exercise. Exercise for the pregnant mother diverts blood to the muscles and skin, creating a short-term hypoxic environment. Hypoxia in the placenta also activates HIF-1 and VEGF and enhances angiogenesis (4, 120). Consistent with these results, other regulatory factors such as VEGF, VEGFR, and hypoxia-inducible factor 1 (HIF-1) increase significantly with exercise in mice (121). It has also been reported that exercise in pregnant women may increase angiogenesis by increasing placental growth factor (PlGF) (32). As mentioned before, another cause of hypertension associated with reduced uteroplacental perfusion pressure (RUPP) is an imbalance between proangiogenic and anti-angiogenic (soluble fms-like tyrosine kinase 1 (sFIt-1)) factors (121). While exercise reduces RUPP-induced blood pressure by lowering sFIt-1 and increasing the VEGF factor and VEGF/sFIt-1 ratio. Hence, the positive effects of exercise on angiogenic balance in RUPP mice are confirmed by endothelial tube formation. Thus, exercise before and during pregnancy reduces hypertension, angiogenic imbalance, and oxidative stress induced by placental ischemia in RUPP mice (122). In addition, muscles stimulate AMPK and VEGF during high-volume exercise and lead to amelioration of preeclampsia during pregnancy. Therefore, exercise during pregnancy improves placental function (i.e., the ratio of fetal weight to placental weight) in both normal and RUPP pregnancies (32, 123). Although AMPK phosphorylation is suppressed as a result of maternal obesity, increased levels of AMPK phosphorylation and apelin protein in the placenta after exercise indicate a possible role of apelin angiogenesis mediated by AMPK in the placenta. Thus increase in AMPK phosphorylation in trained mice indicates nutrient/oxygen exchange in the placenta (4).

## Possible roles of apelin and exercise to control vascular tone

Apelin and APJ are found in many placental cells such as endothelial cells and embryonic artery smooth muscle cells. Apelin appears to affect placental vascular tone and the exchange of oxygen and nutrients between mother and fetus (1). In addition, in the placenta, an organ without autonomic nerves, local control of vascular tone is critical to maintaining fetal growth because endothelial dysfunction is common due to an imbalance between the synthesis of vasodilator and vasoconstrictor molecules in the systemic circulation and placenta (124). Apelin, through the L-arginine/eNOS/NO pathway, increases vasodilation, heart contraction, angiogenesis, and suppression of aortic inflammation (39, 89, 115, 125) (**Figure 2**). eNOS converts L-arginine to NO molecule (39, 115). The NO released from endothelial cells is transported to adjacent vascular smooth muscle cells (VSMCs), where it causes cGMP production and Ca2+ uptake into intracellular calcium stores. As a result, Ca2+ is reduced, causing VSMC relaxation and vasodilation (126). NO is a strong vasodilator, a blood pressure regulator, and an anti-atherogenic, and prevents cell adhesion and platelet aggregation. In the placenta, eNOS is highly expressed in syncytiotrophoblast, villi endothelium, and macrophages (39, 115). Studies have shown that decreasing levels of apelin, endothelial nitric oxide synthase (eNOS), and nitric oxide (NO) contribute to the pathogenesis of preeclampsia. In preeclampsia, oxidative markers such as malondialdehyde (MDA) increase significantly, and levels of NO, apelin, and eNOS decrease significantly. Mean arterial pressure is negatively correlated with apelin and NO, and MDA is positively correlated with mean arterial pressure (115). Therefore, the decrease in mean serum levels of apelin and eNOS is normal in preeclamptic women, and it is negatively correlated with mean arterial pressure (39, 115, 125, 127, 128). In contrast, rats treated with apelin improve the symptoms of preeclampsia because it significantly increases the expression of eNOS in the placenta and the levels of NO and eNOS in the serum, all of which decrease preeclampsia (1, 39, 129). There is evidence that after the occurrence of preeclampsia, the level of maternal apelin to dilate blood vessels to cope with maternal hypertension increases (1). In addition, intravenous injection of apelin-13 into pregnant women increases the transfer of glucose from mother to fetus through the placenta. Due to the lack of change in the expression of major placental glucose transporters such as GLUT1 and GLUT3, apelin seems to exert its effect by increasing the dilation of placental arteries. Apelin probably absorbs glucose in the muscle through NO-induced vasodilation (1). Shear stress due to moderate exercise is one of the most important mechanisms to improve vascular function in the placenta through the synthesis of NO, which is caused by eNOS and VEGF (124). Exercise during pregnancy in the human placenta leads to a 2-fold increase in eNOS and a 4-fold increase in NO, as well as a 6% decrease in O2 levels and a 26% decrease in H2O2 (130). On the other hand, chronic endurance training improves blood pressure by increasing mRNA expression and eNOS phosphorylation and reducing oxidative stress (126). Exercise-related mechanisms appear to involve the high expression of eNOS and NO because L-arginine and NO increase after 12 weeks of exercise and contribute to the beneficial effects of exercise on high blood pressure in humans (131). Exercise has been reported to be effective in preventing the onset of preeclampsia because 6 weeks of exercise through small heat shock proteins is involved in oxidative stress and apoptosis and facilitates eNOS-mediated NO synthesis through the larger HSPs (122). Since recent studies have been on animal models, future research with scientific guidelines on humans seems necessary.

The apelin system is associated with other effective cardiovascular systems such as Angiotensin (ACE) (32). Angiotensin-converting enzyme (ACE), angiotensin II (Ang II), and angiotensin 1 receptor (AT1) act as vasoconstrictors, cell proliferation, limb hypertrophy, sodium retention, and aldosterone release. While the enzyme converter angiotensin 2 (ACE2), angiotensin- (1–7), and Mas receptor are involved in vasodilation, anti-proliferation, anti-hypertrophy, cardiac protection, and protective measures (132, 133). Activation of AT1 receptors stimulates apelin secretion through Ca2+-dependent pathways, protein kinase C, and MAPK kinase, while activation of AT2 receptors inhibits apelin secretion through cAMP-and-cGMP-dependent pathways (134). Apelin is also detected by ACE2 (135) and the placenta can participate in apelin clearance by increasing ACE2 expression and helping to reduce maternal apelin levels at term (1). In preeclampsia, a decrease in apelin/APJ is associated with an increase in ACE2 expression because apelin is metabolized by ACE2 (27, 32, 136). Angiotensin II reduces the release of apelin from the human placenta (1). Ang II and apelin have opposite effects on the regulation of blood pressure, vascular tone, inflammation, and fluid homeostasis (27). Apelin inhibits the increase in cytosolic calcium and vasoconstriction induced by Ang II, which in turn contributes to vasodilation (27, 32). After treatment with apelin, the incidence of preterm delivery, cesarean section, and asphyxia are reduced (137). While positive regulation of Ang II and Ang II type 1 receptor (AT1R) is known as a vasoconstrictor and pro-inflammatory peptides in the placenta in women with preeclampsia (27). Although angiotensin II receptors increase at preeclampsia but decrease in trained mice (138). In rats, decreased inflammatory cytokines and angiotensin II receptor type 1 with high-volume exercise confirm this finding (139). Hence, exercise can stimulate the ACE2-Ang- (1–7)-Mas axis in parallel with inhibiting the ACE-Ang II-AT1 pathway. Activation of the ACE2-Ang- (1–7)-MAS receptor axis may play a role in the beneficial effects of physical exercise (133, 140) (**Figure 2**). Modulation of RAS appears to be a key mechanism in the development of preeclampsia, which can be altered by exercise to prevent preeclampsia (138, 141). In addition, pregnant women without physical activity have the same risks as obesity for preeclampsia (14). Epidemiological studies on exercise during pregnancy show a reduction in the incidence of obesity complications such as gestational diabetes, gestational hypertension, fetal growth retardation, and preeclampsia (32, 142, 143). Finally, current treatment guidelines emphasize the role of physical activity in treating high blood pressure because exercise lowers blood pressure in 75% of people with high blood pressure. Low to moderate-intensity exercise seems to help lower blood pressure in people with high blood pressure (113).

## Possible role of apelin in cardiac hypertrophy in pregnancy

Pregnancy is a unique period in a woman’s life with anatomical and physiological changes to increase the fetal metabolic demand. Hence, the cardiovascular system must be adapted to meet the needs of the fetus and the enlarged uterus (117). In pregnancy, physiological changes in the heart are proportional to an increase in factors such as PGC-1α, VEGF, angiogpietin1, and fibroblast growth factor 2 (FGF-2). Thus, cardiac adaptations due to pregnancy are more like exercise-induced physiological hypertrophy than pathological hypertrophy (117, 144). Under physiological hypertrophy conditions, heart function is either normal or increases due to a proportional increase in chamber size and wall thickness (11). Pathological cardiac hypertrophy is associated with decreased heart function and heart failure. Volumetric and pressure overload initially change the morphometry of the chamber. This change can be concentric hypertrophy (further increase in wall thickness with small cavities) or eccentric hypertrophy (large ventricular cavities with thin walls) (11). The apelin/APJ system plays an important role in cardiac hypertrophy and cardiovascular disease (89). Early studies show that APJs have apelin-independent functions for heart development. For example, fetuses carrying APJ mutations do not have a heart, and APJ knockout mice show fetal death due to vascular abnormalities, hearts with weak rings, and ventricular abnormalities. Most of the live fetuses also show abnormal vasculature, abnormal myocardium formation, and ventricular wall growth in adulthood (1). There is APJ in the early stages of gastrulation and during the later stages of development, while apelin expression begins only at the end of gastrulation. In general, APJ is very important for the early growth of the heart and apelin is vital for the early growth of the placenta and the formation of the fetal cardiovascular system (1).

Cardiac apelin is ​​significantly reduced in heart failure and is regulated directly by the Ang II-AT1R system. Ang II-induced hypertension gradually puts extra pressure on the heart, leading to pathological hypertrophy. Ang II-treated mice show an increase in left ventricular mass (LV), cardiomyocyte cross-section, and atrial natriuretic factor (ANF) expression. Transforming growth factor beta1 (TGF-β1) may contribute to Ang II-induced cardiac pathological hypertrophy. In contrast, the treatment of hypotension with an angiotensin-converting enzyme inhibitor or angiotensin receptor antagonist significantly reduces cardiac hypertrophy. Inhibition of the renin-angiotensin system may have beneficial effects, at least in part, through the repair of the cardiac apelin system. Apelin/APJ overexpression reduces the increased cardiac hypertrophy induced by Ang II, TGF-β, and oxidative stress (145). Apelin, on the other hand, can increase ACE2 levels in defective hearts and metabolize Ang II to produce the beneficial heptapeptide Ang- (1–7) as an anti-cardiac hypertrophy agent. Intraperitoneal apelin injection lowers blood pressure in hypertensive mice by inhibiting the renin-angiotensin system. As a result, apelin may activate the ACE2/Ang- (1–7) axis to reduce cardiac hypertrophy. Overexpression of apelin eliminates Ang II-induced cardiac hypertrophy by reducing atrial natriuretic peptide (ANP) protein content in cardiomyocytes by reducing cell size. However, apelin administration has been reported to cause non-pathological cardiac hypertrophy, and apelin/APJ could be a promising therapeutic target for cardiac hypertrophy (146). While specific inhibitors of PI3k, Akt, and ERK1/2 factors reverse the effects of apelin on the diameter, volume, and protein content of cardiomyocytes. Hence the PI3k-Akt-ERK1/2-p70S6K pathway is involved in rat myocardial hypertrophy caused by apelin (147). Serum apelin is significantly lower in patients with left ventricular hypertension than in normal individuals. In mice and humans, there is a direct correlation between plasma apelin and left ventricular mass index and between apelin expression and APJ in the myocardium. However, in mice with diabetic hypertension, an inverse correlation was observed between plasma apelin and myocardial expression. This means that apelin/APJ expression decreases in the myocardium, while plasma apelin increases in left ventricular hypertrophy. Due to the inotropic properties and vasodilation induced by apelin, plasma apelin enhancement appears to be a compensatory mechanism for maintaining cardiac output in rats with hypertensive overload or diabetic cardiomyopathy (148). There is evidence that a high-fat diet leads to increased ER stress (increased Bip and CHOP levels), and increased intracellular Ca2+ in the heart. The calcineurin-NFAT3 signaling pathway is then activated, leading to cardiac hypertrophy. While with the administration of apelin significantly reduces this pathway (89). Oxidative stress is an important factor in the development of cardiac hypertrophy by the overproduction of ROS and decreased cardiac antioxidant capacity. ROS is involved in cardiac pathological hypertrophy through inflammatory agents such as Ang II, TNF-α, leptin, and endothelin-1. In contrast, apelin significantly inhibits ROS production and increases antioxidant capacity by catalase activity in neonatal cardiomyocytes (149). Thus, apelin/APJ can inhibit pathological cardiac hypertrophy associated with oxidative stress.

On the other hand, exercise can improve myocardial morphology and heart function, leading to hypertrophy of the athlete’s heart. Moderate-intensity exercise (60 to 70% VO2max for 8 weeks) significantly increases PI3K/Akt, mTOR, and p70S6K, but does not show pathological damage to the myocardium. While long-term high-intensity exercise (80 to 85% VO2max) causes cardiac hypertrophy with heart damage, which carries the risk of pathological changes (150). Akt as an axial regulator increases physiological hypertrophy as opposed to pathological hypertrophy through exercise. Swimming exercise in mice lacking Akt disrupts the cardiac growth response (151, 152). It is reported that IGF-1, PI3K, Akt, and mTOR signaling are also involved in exercise-induced cardiac hypertrophy (153). Thus, these results suggest that apelin/APJ and exercise may prevent pathological cardiac hypertrophy by activating the PI3K/Akt/mTOR/p70S6K and PI3k-Akt-ERK1/2-p70S6K pathways. Pathological hypertrophy is induced by prolonged stimulation of pro-inflammatory cytokines such as interleukin IL-1β, IL-6, TNF-α, TGF-β1, And NF-kB. These factors are strongly associated with increased fibrosis. In contrast, the hearts of athlete animals do not increase pro-inflammatory cytokines, and instead, significantly increase the anti-inflammatory cytokine IL-10 (11). These results show the difference between pathological and physiological hypertrophy. However, hypertrophy caused by pregnancy and exercise is both physiological hypertrophies. In mid-pregnancy, Akt phosphorylation and its downstream targets, such as GSK3β, mTOR, and p70S6, increase significantly. As a result, cardiac adaptation during mid-pregnancy is similar to the response to exercise (117). As a result, it seems that exercise through physiological hypertrophy and reduction of inflammatory factors can be useful to protect the heart of pregnant women. Hence, the American Congress of Obstetrics and Gynecology, the centers for disease control, and the American College of Sports Medicine recommend physical activity during pregnancy. These guidelines state that women should exercise at a moderate intensity for at least 30 minutes most days of the week if not all days of the week (117).

## Conclusion

The prevalence of maternal obesity during pregnancy is associated with the risk of metabolic diseases and high birth weight. Overweight and obesity in pregnant women predispose their children to obesity and metabolic diseases in the future (2, 5, 40–42). Obesity is associated with WAT hypertrophy and BAT apoptosis (41, 49). Environmental factors such as active lifestyle, and type of nutrition such as apelin supplementation can lead to BAT activity and thermogenesis (41). Apelin supplementation and exercise (45 to 65% VO2max) during pregnancy increase BAT proteins such as Cidea, Elovl3, UCP1, PRDM16, and PGC-1α in males and female fetuses (5, 22). UCP1 dissipates energy stored in the mitochondrial electrochemical gradient in the form of heat, and PRDM16 positively regulates many of the genes involved in BAT production (41). Released fatty acids are the primary energy source for UCP1-mediated thermogenic (62). Elevated UCP1 and PRDM16 in the offspring of athletic mothers are associated with oxygen consumption (5). Increased levels of VO2 and VCO2 in the offspring of athlete mothers indicate an increase in carbohydrate oxidation and are associated with an increase in weight and BAT temperature. While WAT weight is reduced and indicates an improvement in fetal growth and metabolism in the offspring of athlete mothers (5). Thus, exercise and consumption of apelin by the pregnant mother regulates glucose and fat metabolism, oxidative phosphorylation, thermogenesis, mitochondrial biogenesis, and growth of fetal brown fat and prevents the differentiation of BAT into WAT (5, 68).

Apelin also increases pancreatic islet cell mass and beta-cell insulin content (86). Apelin has a glucose-lowering effect with increased glucose utilization in skeletal muscle and adipose tissue of normal and obese insulin-resistant mice (34). Apelin increases glucose uptake through the PI3K, Akt, PKC, and MAPK factors into adipocytes and can increase the transport of GLUT4 from the cytoplasm to the plasma membrane (56, 87). In diabetes or obesity with hyperinsulinemia, increased apelin is a compensatory mechanism in which it not only inhibits pancreatic secretion but also leads to insulin sensitivity and glucose uptake into insulin-independent muscle tissue and then leads to a decrease in apelin levels (31, 88). Exercise-induced apelin secretion by activating AMPK stimulates the expression of GLUT4 gene in muscle (93), and then activates mitochondrial biogenesis. The offspring muscle in athlete mothers expresses higher levels of the PGC-1α1/4 isoform and increases oxidative muscle fibers in children. Oxidative muscle fibers are very efficient in the use of glucose and fatty acids (15, 91). Hence the useless energy consumption in the muscle increases (2, 42). In addition, boys and girls of athlete mothers show better glucose tolerance than children in the control group, which is associated with lower fasting blood sugar and insulin levels in the children of athlete mothers (5). Hence, exercise and apelin are known as regulators of energy metabolism and have anti-obesity and anti-diabetic properties (52).

To prevent preeclampsia, exercise in the pregnant mother directs blood to the muscles and skin, creating a short-term hypoxic environment. Hypoxia activates HIF-1, VEGF, and VEGFR and increases angiogenesis (121). Exercise and apelin, through the L-arginine/eNOS/NO pathway, increase vasodilation, angiogenesis, and suppression of inflammation (39, 89, 115, 125). Exercise can stimulate the ACE2-Ang- (1–7)-Mas axis in parallel with inhibiting the ACE-Ang II-AT1 pathway (133). The activity of RAS appears to be a key mechanism in the development of preeclampsia that can be altered by exercise to prevent preeclampsia (138, 141). Moderate-intensity exercise (60 to 70% VO2max) and apelin/APJ may prevent pathological hypertrophy by activating the PI3K/Akt/mTOR/p70S6K, PI3k-Akt-ERK1/2-p70S6K pathways and the anti-inflammatory cytokine IL-10. While pathological hypertrophy is induced by long-term stimulation of pro-inflammatory cytokines such as interleukins IL-1β, IL-6, TNF-α, TGF-β1, and NF-κB (11, 150). As a result, it seems that exercise through these pathways can be beneficial in protecting the heart of pregnant women.

## Future prospects

Since the present study studies are more than animal samples, future research should pay more attention to human samples. The use of scientific guidelines for maternal and fetal health should be considered. Future research should pay special attention to the variables of duration and intensity of training. Due to physiological changes during the first, second, and third trimesters of pregnancy, research should consider these time variables.

## Author contributions

The author confirms being the sole contributor of this work and has approved it for publication.

## Conflict of interest

The author declares that the research was conducted in the absence of any commercial or financial relationships that could be construed as a potential conflict of interest.

## Publisher’s note

All claims expressed in this article are solely those of the authors and do not necessarily represent those of their affiliated organizations, or those of the publisher, the editors and the reviewers. Any product that may be evaluated in this article, or claim that may be made by its manufacturer, is not guaranteed or endorsed by the publisher.

## Abbreviations

BMI, body mass index; APJ, apelin receptor; AT1, angiotensin II receptor; WAT, white adipose tissue; BAT, brown adipose tissue; TG, triglycerides; FFA, free fatty acids; C/EBPβ, CCAAT-enhancer-binding protein; PPARγ, peroxisome-proliferator-activated receptor-γ; PRDM16, PR domain-containing protein 16; PGC1α, peroxisome proliferator-activated receptor-gamma coactivator 1-alpha; UCP1, uncoupling protein 1; AKT, protein kinase B; PKA, protein kinase A; cAMP, cyclic adenosine monophosphate; SNS, central nervous system; HSL, hormone-sensitive lipase; N, norepinephrine; PKC, protein kinase C; RyR1, ryanodine receptor; PLP, phospholamban; SLN, sarcolipin; UCP3, uncoupling protein 3; GLUT4, glucose transporter 4; IRE1α, inositol-requiring enzyme 1α; JNK, Jun N-terminal Kinase; PI3K, phosphatidylinositol 3-kinase; AMPK, AMP-activated protein kinases; ACC, acetyl CoA carboxylase; CoA, malonyl-coenzyme A; mtDNA, mitochondrial DNA; CVD, cardiovascular disease; RAS, renin-aldosterone-angiotensin; sFIt-1, soluble fms-like tyrosine kinase 1; VEGFR, vascular endothelial growth factor receptor; HIF-1, hypoxia-inducible factor 1; RUPP, uteroplacental perfusion pressure; VSMCs, vascular smooth muscle cells; eNOS, nitric oxide synthase; NO, nitric oxide; MDA, malondialdehyde; VEGF, vascular endothelial growth factor; ACE, Angiotensin-converting enzyme; Ang II angiotensin II; ACE2, enzyme converter angiotensin 2; FGF-2, fibroblast growth factor 2.
